# Mechanism of N-0385 blocking SARS-CoV-2 to treat COVID-19 based on molecular docking and molecular dynamics

**DOI:** 10.3389/fmicb.2022.1013911

**Published:** 2022-10-18

**Authors:** Jun-Feng Cao, Xingyu Yang, Li Xiong, Mei Wu, Shengyan Chen, Chenyang Xiong, Peiyong He, Yonghua Zong, Lixin Zhang, Hongjiao Fu, Yue Qi, Xiran Ying, Dengxin Liu, Xiaosong Hu, Xiao Zhang

**Affiliations:** ^1^Clinical Medicine, Chengdu Medical College, Chengdu, China; ^2^University of Tibetan Medicine, Lhasa, China; ^3^Yunnan Academy of Forestry Sciences, Kunming, Yunnan, China; ^4^Chengdu Medical College of Basic Medical Sciences, Chengdu, China

**Keywords:** COVID-19, N-0385, bioinformatics analysis, molecular docking, molecular dynamics

## Abstract

**Purpose:**

2019 Coronavirus disease (COVID-19) has caused millions of confirmed cases and deaths worldwide. TMPRSS2-mediated hydrolysis and maturation of spike protein is essential for SARS-CoV-2 infection *in vivo*. The latest research found that a TMPRSS2 inhibitor called N-0385 could effectively prevent the infection of the SARS-CoV-2 and its variants. However, it is not clear about the mechanism of N-0385 treatment COVID-19. Therefore, this study used computer simulations to investigate the mechanism of N-0385 treatment COVID-19 by impeding SARS-CoV-2 infection.

**Methods:**

The GeneCards database was used to search disease gene targets, core targets were analyzed by PPI, GO and KEGG. Molecular docking and molecular dynamics were used to validate and analyze the binding stability of small molecule N-0385 to target proteins. The supercomputer platform was used to simulate and analyze the number of hydrogen bonds, binding free energy, stability of protein targets at the residue level, radius of gyration and solvent accessible surface area.

**Results:**

There were 4,600 COVID-19 gene targets from GeneCards database. PPI, GO and KEGG analysis indicated that signaling pathways of immune response and inflammation played crucial roles in COVID-19. Molecular docking showed that N-0385 could block SARS-CoV-2 infection and treat COVID-19 by acting on ACE2, TMPRSS2 and NLRP3. Molecular dynamics was used to demonstrate that the small molecule N-0385 could form very stable bindings with TMPRSS2 and TLR7.

**Conclusion:**

The mechanism of N-0385 treatment COVID-19 was investigated by molecular docking and molecular dynamics simulation. We speculated that N-0385 may not only inhibit SARS-CoV-2 invasion directly by acting on TMPRSS2, ACE2 and DPP4, but also inhibit the immune recognition process and inflammatory response by regulating TLR7, NLRP3 and IL-10 to prevent SARS-CoV-2 invasion. Therefore, these results suggested that N-0385 may act through multiple targets to reduce SARS-CoV-2 infection and damage caused by inflammatory responses.

**Graphical Abstract:**
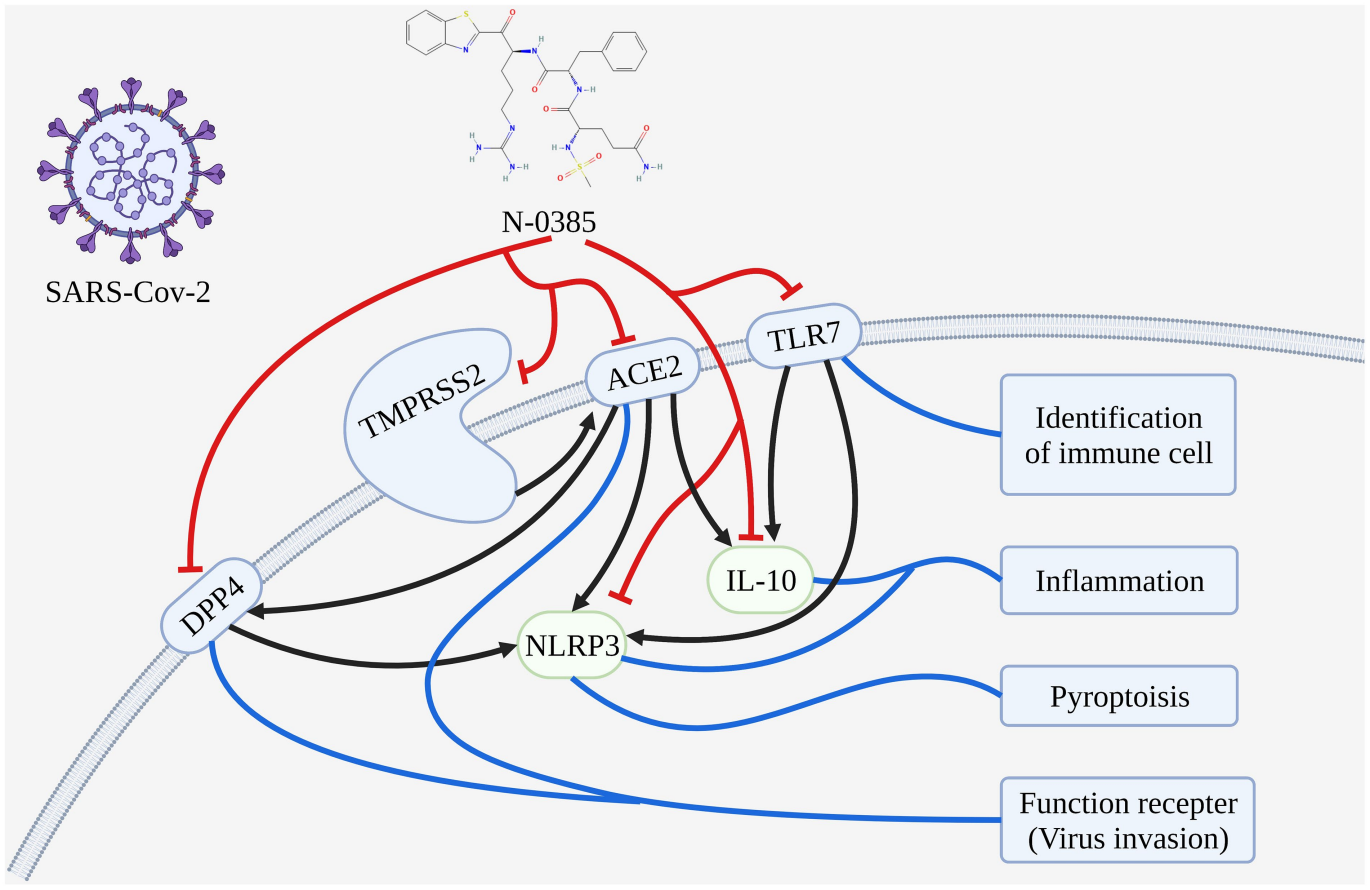
The mechanisms analysis of N-0385 blocking SARS-CoV-2 infection in the treatment of COVID-19.

## Introduction

COVID-19 is a malignant infectious disease caused by SARS-CoV-2 infection, it is still an ongoing life-threating event. SARS-CoV-2 belongs to the genus beta coronavirus (Perlman and Netland, 2009; Su et al., 2016; Rai et al., 2021). It is found that SARS-CoV-2 is transmitted person to person mainly through inhalation or contact with infectable droplets, with an incubation period of 2–14 days (Lin et al., 2020; Liu et al., 2020; Rohit et al., 2020). The SARS-CoV-2 spike protein binding to ACE2 can promote viral infection of cells (Khateeb et al., 2021). The clinical symptoms of COVID-19 include respiratory syndrome (such as: shortness of breath, cough, dyspnea, fever and viral pneumonia; Cheng et al., 2020; Huang et al., 2020). Studies have found that exacerbation of COVID-19 disease was associated with secondary systemic, inflammatory responses and cytokine storms. Critically ill patients may progress to ARDS, septic shock, multi-organ failure and ultimately death (Rai et al., 2021). Current research has found that there are many key protein targets that may play a key role in COVID-19. Transmembrane Serine Protease 2 (TMPRSS2) is a serine protease family protein, and TMPRSS2 can facilitate virus (such as: SARS-CoV-2, HCoV-229E and MERS-CoV) entry into host cells through proteolytic cleavage and activation of viral envelope glycoproteins (Koch et al., 2021). Angiotensin Converting Enzyme 2 (ACE2) is a metalloproteinase, and the main physiological role of ACE2 is to regulate vasoconstriction and blood pressure (Lan et al., 2020). Some studies have made certain that ACE2 is the receptor for severe acute respiratory syndrome coronavirus 2 (SARS-CoV-2; Beyerstedt et al., 2021). Toll Like Receptor 7 (TLR7) is a member of the Toll-like receptor family. TLR7 plays a key role in recognizing SARS-CoV-2 and initiating the development of an early antiviral immune response (Solanich et al., 2021). Dipeptidyl Peptidase 4 (DPP4) is a serine exopeptidase that selectively degrades a variety of substrates (including incretin hormones, growth factors and cytokines; Nargis and Chakrabarti, 2018). Study has been reported that there may be a tight interaction between the COVID-19 spike glycoprotein and DPP4 (Vankadari and Wilce, 2020). Interleukin 10 (IL-10) is a key anti-inflammatory mediator capable of protecting the host from damage caused by over-activated inflammatory responses (Wang et al., 2019; Saraiva et al., 2020). It has been demonstrated that there is a dramatic increase in IL-10 expression in the COVID-19 cytokine storm, which is thought to be a fundamental function for suppressing inflammation (Lu et al., 2021). NLR Family Pyrin Domain Containing 3 (NLRP3) promotes inflammasome formation, and NLRP3 activates MAPK and NF-κB signaling cascades to regulate innate and adaptive immune systems (Zhen and Zhang, 2019).

In current clinical treatment, clinical drugs and vaccines remain the mainstay of prevention and treatment of COVID-19, but vaccine approaches have little efficacy against new variants of the new crown. Current clinical drugs include antivirals (Ritonavir, Lopinavir and Raltegravir), antimalarials (Hydroxychloroquine and Chloroquine) and anti-inflammatory corticosteroids (Dexamethasone and Prednisolone; Khan and Al-Balushi, 2021). However, the effectiveness of current clinical therapeutic agents is not satisfactory. Although clinical improvement has been reported in patients with neoconjunctivitis after the use of Lopinavir/Ritonavir (Lim et al., 2020). Li showed little benefit of lopinavir/ritonavir in improving clinical outcomes in patients hospitalized with mild/moderate COVID-19 (Li et al., 2020), and these drugs triggered a minority of gastrointestinal adverse events. Although chloroquine and hydroxychloroquine have been effective in clinical treatment as anti-malarial drugs, they are inherently toxic, especially to the eyes and heart (Gautret et al., 2020; Wang et al., 2020). Monupivir has shown significant benefit in the treatment of mild SARS-CoV-2, but the therapeutic role of monupivir with moderate to severe COVID-19 is unclear (Singh et al., 2021). An *in vitro* study showed that atazanavir inhibited the replication of SARS-CoV-2 more than lopinavir (Fintelman-Rodrigues et al., 2020). In addition, it has been shown that plasma or clean monoclonal antibodies developed from fully recovered COVID-19 patients can be offered as therapeutic agents to new COVID-19 patients (Ashour et al., 2022). Long-term administration of chloroquine and hydroxychloroquine can lead to irreversible harm (such as: retinopathy and cardiomyopathy; Motarjemizadeh et al., 2015; Chatre et al., 2018). The role of anti-inflammatory corticosteroids in the treatment of serious infections has been in controversy, and there are limited data on the treatment of COVID-19. Although drug repurposing can be used as a contingency plan in the treatment of diseases, the results of therapies used to treat patients with COVID-19 are highly controversial due to the different study populations (Al-Karmalawy et al., 2021). Therefore, there is a lack of drugs that can effectively hinder viral infection with fewer side effects in the clinical treatment of COVID-19.

N-0385 is a Transmembrane Serine Protease 2 (TMPRSS2) targeted peptidomimetic compound. N-0385 is also known as Ms-QFR-Kbt. The molecular weight of N-0385 is 644.77, and the molecular formula of N-0385 is C_28_H_36_N_8_O_6_S_2_ (Shapira et al., 2022). TMPRSS2 is a key protease for the entry of SARS-CoV-2 into cells, and TMPRSS2 is able to cleave the SARS-CoV-2 spike protein to initiate virus invasion and infection of cells (Hoffmann et al., 2020; Zhang et al., 2020; Kishimoto et al., 2021). Study found that N-0385 could effectively inhibit SARS-CoV-2 infection in human lung epithelial cells at the half-maximal inhibitory concentration (IC50) of 12.3 ± 1.9 nM. Interestingly, N-0385 was able to target TMPRSS2 to effectively inhibit SARS-CoV-2 infection in Calu-3 cells, these results indicated that N-0385 was a highly efficient inhibitor of TMPRSS2 and could block SARS-CoV-2 entry into cells. Surprisingly, when K18-hACE2 mice were infected with SARS-CoV-2B.1.617. N-0385 treatment completely prevented SARS-CoV-2-induced mortality and significantly prevented weight loss, pulmonary pathology and viral infection (Shapira et al., 2022). This finding deduces that N-0385 can serve as an antiviral agent against pan-mutated viruses. Therefore, N-0385 will provide a proven protocol for the early treatment of COVID-19 and the responding to emerging variant SARS-CoV-2.

However, the research on N-0385 is just beginning, and more in-depth studies on N-0385 are lacking. Therefore, this study investigates the mechanism of N-0385 treatment COVID-19 through molecular docking and molecular dynamics. Bioinformatics were used to screen for COVID-19 core targets. Gene Ontology (GO), Kyoto Encyclopedia of Genes and Genomes (KEGG) and Protein–Protein Interactions (PPI) were used to analyze gene targets and explore their mechanisms of action and potential pathways. The molecular system motions were used to simulate the result of complex binding from cellular level to chemical moiety level. Molecular docking was used to determine the affinity of the small molecule N-0385 and protein targets. Molecular dynamics was used to simulate the stability of the binding complex. Therefore, the mechanism of N-0385 treatment COVID-19 in this study will facilitate research and clinical applications related to N-0385.

## Materials and methods

### Acquisition and screening disease gene targets

In this study, we used “COVID-19” and “SARS-CoV-2” as keywords to obtain disease gene targets from the GeneCards database. The relevance score ≥ 5 was used as a threshold to screen COVID-19 related genes targets from GeneCards database, the relevance score is a comprehensive evaluation of the association of genes with the studied diseases.

### Protein–protein interaction network construction

The STRING database was used to analyze protein–protein interactions of COVID-19 and construct the PPI network. We imported COVID-19 related protein targets obtained from GeneCards database into Cytoscape 3.7.1 and STRING database for analysis (Rastelli et al., 2010). The network topology parameters were analyzed by Cytoscape 3.7.1, and the core protein targets were filtered according to the criteria of node degree value and median center value greater than the average value.

### Gene target enrichment analysis

We used the related genes targets in the DAVID database for Gene Ontology (GO) and Kyoto Encyclopedia of Genes and Genomes (KEGG) enrichment analysis. GO enrichment was used to obtain biological information about gene targets in biological processes (BP), cellular components (CC) and molecular functions (MF). KEGG pathway enrichment was performed by enriching the signaling pathways involved in the related gene targets. Gene targets screening was performed at *p* < 0.05 to analyze the main signaling pathways and biological processes of COVID-19. Omicshare tool platform was used to visualize the results of GO enrichment and KEGG enrichment (Cao et al., 2022b).

### Molecular docking and validation of molecular docking

Molecular docking was used to study the molecular affinity of the small molecule N-0385 to the relevant protein targets of COVID-19. The protein crystal structures were downloaded from the PDB database, and 3D structures of small molecules were downloaded from the PUBCHEM database. We performed the molecular docking work by employing AutoDock Vina 1.1.2 software (Kim et al., 2016). Prior to docking, PyMol 2.5 was used to process all receptor proteins (including removal of water molecules, salt ions and small molecules). ADFRsuite 1.0 was used to convert all processed small molecules and receptor proteins into the PDBQT format required for docking with AutoDock Vina 1.1.2 (Ravindranath et al., 2015). The docked conformation with the highest molecular docking score was considered to be the binding conformation for subsequent molecular dynamics simulations. We analyzed and compared the binding site poses, chemical bond lengths and chemical bond angles of the original crystal ligand to the protein by re-docking the original crystal ligand and the protein using the original crystal ligand of the protein target as a positive reference. Finally, the consistency of the binding mode can indicate the correctness of the molecular docking scheme. And the value of molecular docking score was greater than 6.5 among the target proteins and the small molecule N-0385, it was considered to be tightly bound (Cao J. et al., 2022).

### Analysis of proteins localized in the cell membrane and screening of core protein targets

Analysis of current research results on small molecule N-0385, we found that the small molecule N-038 may act mainly by acting on key proteins (such as: TMPRSS2) in the cellular cytosol thus preventing SARS-CoV-2 from invading cells to reduce the infection (Shapira et al., 2022). Therefore, this study will focus on the small molecule N-0385 and the proteins expressed on the cell membrane involved in SARS-CoV-2 infected cells. We imported COVID-19 related targets obtained from the GeneCards database into the COMPARTMENT database. The COMPARTMENT database is a database for describing and analyzing protein localization at subcellular level. Proteins localized at the cell membrane with Z-score > 4 were considered highly expressed at the cell membrane. The protein targets that bound tightly to the small molecule N-0385 and the protein targets that were localized at the cell membrane were intersected. Therefore, we obtained core protein targets that bind tightly to the small molecule N-0385 and may be involved in SARS-CoV-2 infected cells.

### Molecular dynamics

In this study, molecular dynamics simulations were further validated the stability of the complex formed by the core target protein and the small molecule N-0385, which was screened by combining bioinformatics analysis and molecular docking results.

Molecular dynamics (MD) relies on Newtonian mechanics to simulate the motion of molecular systems, and the stability of the complex is studied by taking samples from a complex composed of different states of the molecular system and calculating macroscopic properties such as the thermodynamic quantities of the system (Skariyachan, 2022). We used AMBER 18 software for all-atom molecular dynamics simulations (Maier et al., 2015). The ligands were parameterized using a combination of AmberTools18 and ACPYPE51 protocols. The energy of all proteins was minimized by using the most rapid descent method and the conjugate gradient method, and the coordinates and energies of the system are saved every 10 ps. Finally, molecular dynamics simulations were performed for 100 ns for each system under periodic boundary conditions (Larini et al., 2007; Skariyachan et al., 2021). Molecular dynamics simulations included protein-ligand complex root mean square deviation (RMSD), root mean square fluctuation (RMSF), solvent accessible surface area (SASA) and radius of gyration (Rog).

### Molecular Mechanics/Poisson Boltzmann (Generalized Born) Surface Area binding free energy calculation

The Molecular Mechanics/Poisson Boltzmann (Generalized Born) Surface Area (MM-GBSA) method was used to calculate the free energy of binding between proteins and ligands (Rastelli et al., 2010). We used 100 ns molecular dynamics simulations for the calculation. The calculation equation is as follows:


$$\begin{array}{l} \Delta G_{\mathrm{bind}}=\Delta G_{\mathrm{complex}}–\left(\Delta G_{\mathrm{receptor}}+\Delta G_{\mathrm{ligand}}\right)=\Delta E_{\mathrm{internal}}\\ +\Delta E_{VdW}+\Delta E_{\mathrm{elec}}+\Delta G_{\mathrm{GB}}+\Delta G_{SA} \end{array}$$

The non-polar solvation free energy (ΔG_GA_) was calculated based on solvent accessible surface area (SASA) and product of surface tension (γ), ΔG_GA_ = 0.0072 × SASA (Cao et al., 2022a).

## Results

### Acquisition of disease related genes targets and construction of protein interaction network

**Figure 1:**
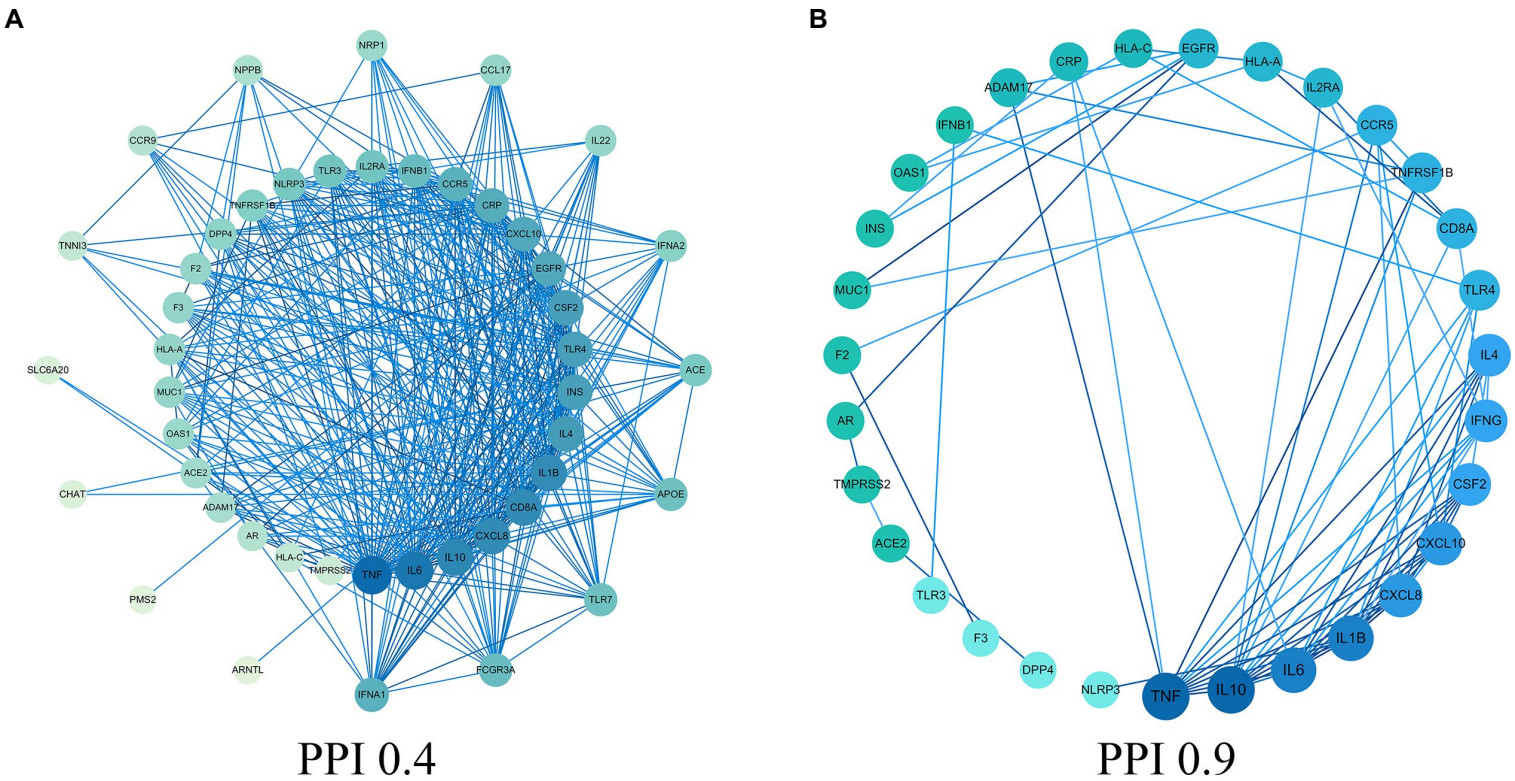
Protein–protein interaction (PPI) network. **(A)** PPI network of protein target. **(B)** PPI network of core protein target (confidence  > 0.9).

In this study, there were 4,600 COVID-19 gene targets. We obtained COVID-19 related gene targets from the GeneCards database based on the relevance score. The relevance score ≥5 was considered as COVID-19 related gene targets, and 51 COVID-19 related gene targets were obtained. The protein interaction network of COVID-19 was constructed by using the STRING database, shown in Figure 1A. The 31 core protein targets (such as: TNF, IL-10, IL6, etc.) were obtained by increasing the confidence score ≥ 0.9. Bioinformatic analysis showed that inflammatory factors (such as: CSF2, IFNG, and CXCL8) occupied the majority of the core protein interaction network, and this is followed by growth factors that affect cell growth and receptor proteins involved in SARS-CoV-2 infected cells. Moreover, the factors involved in the regulation of acute inflammation (such as: IL-1β, IL-10 and IL6) were very closely linked to other targets suggesting that acute inflammation and viral infection played a major role in COVID-19, and the core protein interaction network was reconstructed by using the 31 core protein targets, shown in Figure 1B.

### Gene ontology and KEGG enrichment analysis

**Figure 2:**
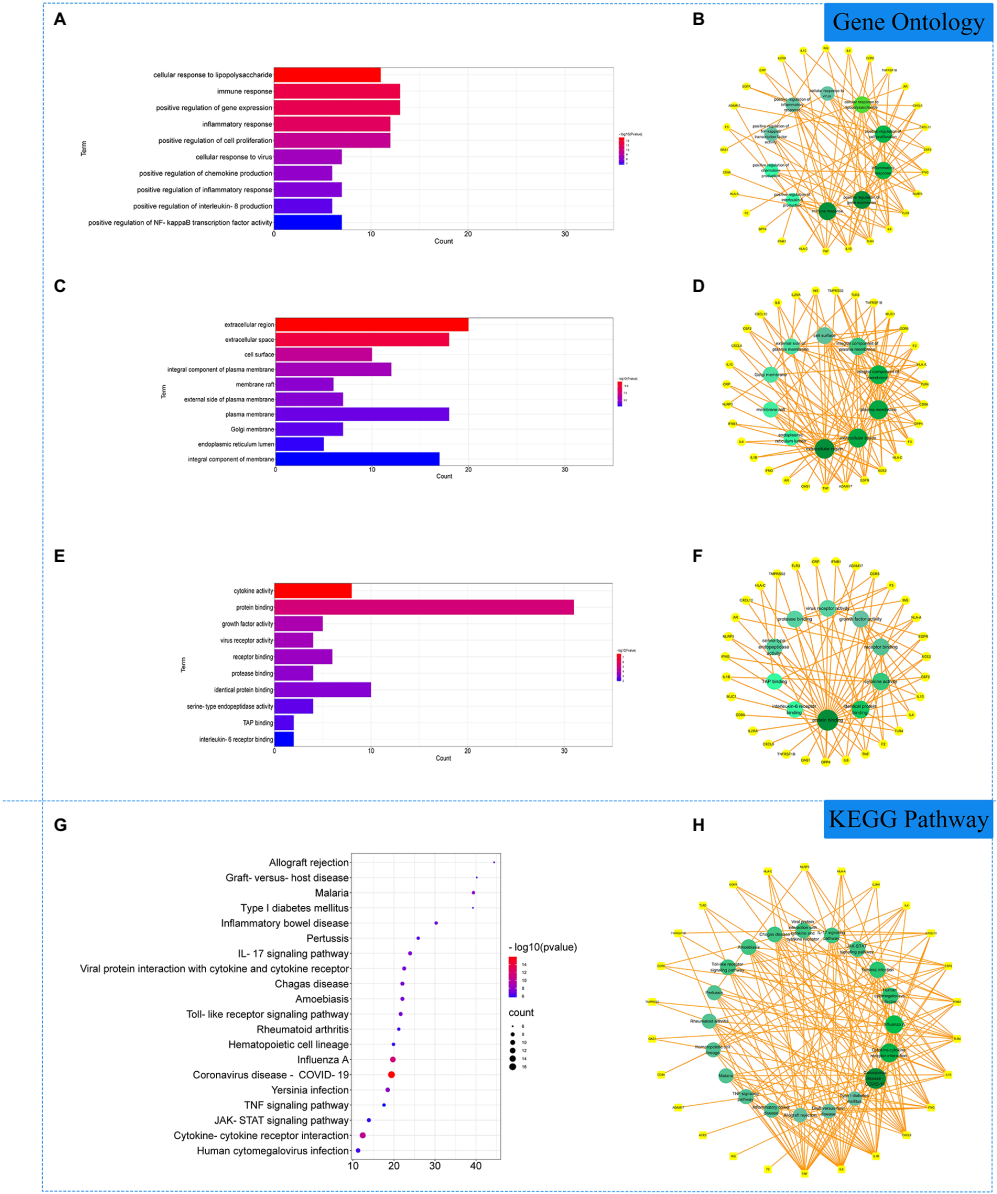
Gene Ontology (GO) and Kyoto Encyclopedia of Genes and Genomes (KEGG) analysis of related genes. **(A)** The top 10 terms in biological processes (BP) were greatly enriched. **(B)** The subnetwork displayed the top 10 BP terms and related genes. **(C)** The top 10 terms in cellular components (CC) were greatly enriched. **(D)** The subnetwork displayed the top 10 CC terms and related genes. **(E)** The top 10 terms in molecular function (MF) were greatly enriched. **(F)** The subnetwork displayed the top 10 MF terms and related genes. **(G)** The top 20 KEGG pathways were showed. **(H)** The subnetworks displayed the top 20 KEGG pathways.

The 51 COVID-19 related gene targets were imported into DAVID database for enrichment analysis. At *p* < 0.05, Gene Ontology (GO) enrichment analysis yielded 256 GO, including 215 biological processes (BP), 22 cellular components (CC) and 19 molecular function (MF). The results showed that biological processes were correlated with cell growth and production of inflammatory factors, mainly involving cellular response to lipopolysaccharide, immune response and positive regulation of gene expression. In cellular component, extracellular region, extracellular space, cell surface accounted for a larger proportion. In molecular functions, cytokine activity, protein binding and growth factor activity were relatively high, shown in Figures 2A–F. KEGG pathway analysis yielded 75 pathways, KEGG enrichment analysis revealed signaling pathways involved in infection and immune response, mainly coronavirus disease—COVID-19, cytokine–cytokine receptor interaction, influenza A and other signaling pathways, shown in Figures 2G,H.

### Screening and analysis of key protein targets

**Figure 3:**
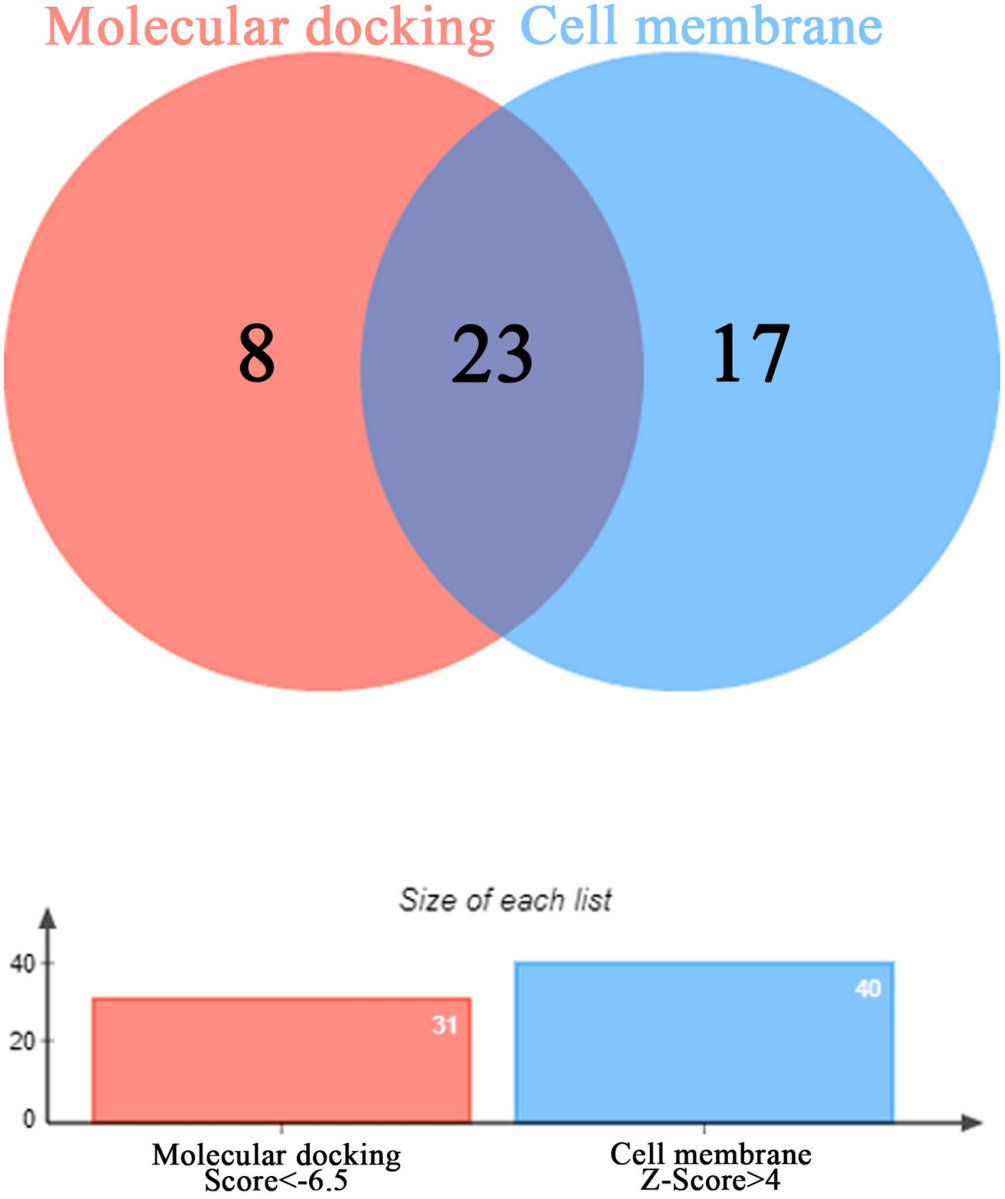
The Venny diagram of intersection targets. The intersection of protein targets highly expressed in cell membranes and protein targets tightly bound to the small molecule N-0385.

The binding energy scores of 51 protein targets (such as: ACE2, INS and TLR3) and the small molecule drug N-0385 were obtained by molecular docking simulations, the molecular docking results are shown in Table 1. The value of molecular docking score was greater than 6.5 among the target proteins and the small molecule N-0385, it was considered to be tightly bound. We obtained 31 protein targets (such as: PMS2, NLRP3 and DPP4) that could form tight bindings to the small molecule N-0385. And we obtained the distribution of the corresponding protein target in the cell by COMPARTMENT database. Proteins localized at the cell membrane with Z-score > 4 were considered highly expressed at the cell membrane. We obtained 40 protein targets (such as: EGFR, ACE2 and TMPRSS2) that were highly expressed in cell membranes. The protein targets that are highly expressed in the cell membrane and those that bind tightly to the small molecule N-0385 were intersected by Venny. The 23 intersecting protein targets (such as: CCR9, ACE2 and TMPRSS2) were those that bound tightly to the small molecule N-0385 and were highly expressed in the cell membrane, shown in Figure 3. Since the results so far have shown that only proteins with both of these characteristics are likely to be the targets of N-0385. We further obtained the scores of these proteins from the GeneCards database, the relevance score ≥ 12 was considered as key protein target. The 6 key protein targets (including: ACE2, TLR7, TMPRSS2, IL-10, NLRP3 and DPP4) were screened by relevance score.

**Table 1:** Results of molecular docking scores.

Target_name	Ligand_name	Docking_score	Target_name	Ligand_name	Docking_score
PMS2	N-0385	−9.6	IFNA2	N-0385	−6.8
MUC1	N-0385	−8.9	INS	N-0385	−6.7
ACE	N-0385	−8.7	IFNG	N-0385	−6.7
CCR5	N-0385	−8.6	FCGR3A	N-0385	−6.7
NLRP3	N-0385	−8.6	ABO	N-0385	−6.6
DPP4	N-0385	−8.4	TLR4	N-0385	−6.5
TAMM41	N-0385	−8.3	IFNA1	N-0385	−6.5
ADAM17	N-0385	−8.2	CD8A	N-0385	−6.5
F2	N-0385	−8.1	CRP	N-0385	−6.5
IL2RA	N-0385	−8.1	IFNB1	N-0385	−6.4
HLA-C	N-0385	−8.1	APOE	N-0385	−6.3
SLC6A20	N-0385	−8	TLR3	N-0385	−6.2
AR	N-0385	−7.9	IL6	N-0385	−6.2
NRP1	N-0385	−7.8	IL-1β	N-0385	−6.1
TNFRSF1B	N-0385	−7.8	CXCL10	N-0385	−6.1
EGFR	N-0385	−7.7	NPPB	N-0385	−6
TLR7	N-0385	−7.7	TNNI3	N-0385	−5.9
ARNTL	N-0385	−7.6	IL22	N-0385	−5.9
HLA-A	N-0385	−7.6	CSF2	N-0385	−5.8
CHAT	N-0385	−7.6	F3	N-0385	−5.8
CCR9	N-0385	−7.4	TNF	N-0385	−5.7
ACE2	N-0385	−7.3	CD99	N-0385	−5.7
IL-10	N-0385	−7.2	IL4	N-0385	−5.5
INPP5E	N-0385	−7.2	CCL17	N-0385	−5.4
TMPRSS2	N-0385	−6.8	CXCL8	N-0385	−5.3
OAS1	N-0385	−6.8			

### Molecular docking

**Figure 4:**
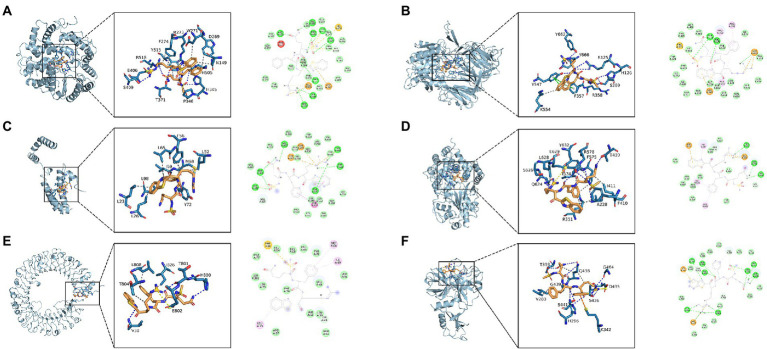
Molecular docking of small molecule N-0385 and protein targets. **(A)** N-0385/ACE2, **(B)** N-0385/DPP4, **(C)** N-0385/IL-10, **(D)** N-0385/NLRP3, **(E)** N-0385/TLR7, **(F)** N-0385/TMPRSS2.

The results indicated that N-0385/ACE2 was mainly maintained by hydrogen bonding and hydrophobic interactions. The small molecule N-0385 interacted with Ser-409, Arg-518, Thr-371, Pro-346, His-345 and Arg-273 on ACE2 protein by hydrogen bonding and with Phe-274, Trp-271, Asp-269, Asn-149 and Tyr-515 by hydrophobic interactions. And N-0385 also interacted with Glu-406 by ionic bonding, shown in Figure 4A. The small molecule N-0385 interacted with Tyr-662, Tyr-666, Arg-125, His-126, Arg-358, Tyr-547 and Ser-209 on DPP4 protein by hydrogen bonding and with Lys-554 by hydrophobic interactions. And N-0385 interacted with Tyr-547 and Phe-357 on DPP4 protein by pi–pi interactions and with Tyr-666 by cationic-pi interactions, shown in Figure 4B. In N-0385/IL-10, the small molecule N-0385 interacted with Leu-23, Leu-26, Tyr-72, Leu-98, Leu-65, Ile-69, Met-68, Phe-56 and Leu52 on IL-10 protein by hydrophobic interactions, shown in Figure 4C. The binding of N-0385/NLRP3 indicated that N-0385 interacted with Ser-626, Glu-624, Ala-228, Tyr-632, Arg-578 and Thr-439 on NLRP3 protein by hydrogen bonding and with Leu-628, Arg-351, Phe-410, Ile-411, Tyr-632 and Ile-574 by hydrophobic interactions. And N-0385 also interacted with Glu-629 by ionic bonding, shown in Figure 4D. The binding of N-0385/TLR7 indicated that N-0385 interacted with Val-38, Thr-801 and His-800 on TLR7 protein by hydrogen bonding and with Thr-804, Leu-808 and Ile-826 by hydrophobic interactions. And N-0385 also interacted with Glu-802 by ionic bonding, shown in Figure 4E. The binding of N-0385/TMPRSS2 indicated that N-0385 interacted with Thr-393, Gln-438, Ser-441, His-296, Ser-436 and Gly-464 on TMPRSS2 protein by hydrogen bonding and with Val-280 and Gln-438 by hydrophobic interactions. And N-0385 also interacted with Asp-435 by ionic bonding, shown in Figure 4F.

### Molecular dynamics results

**Figure 5:**
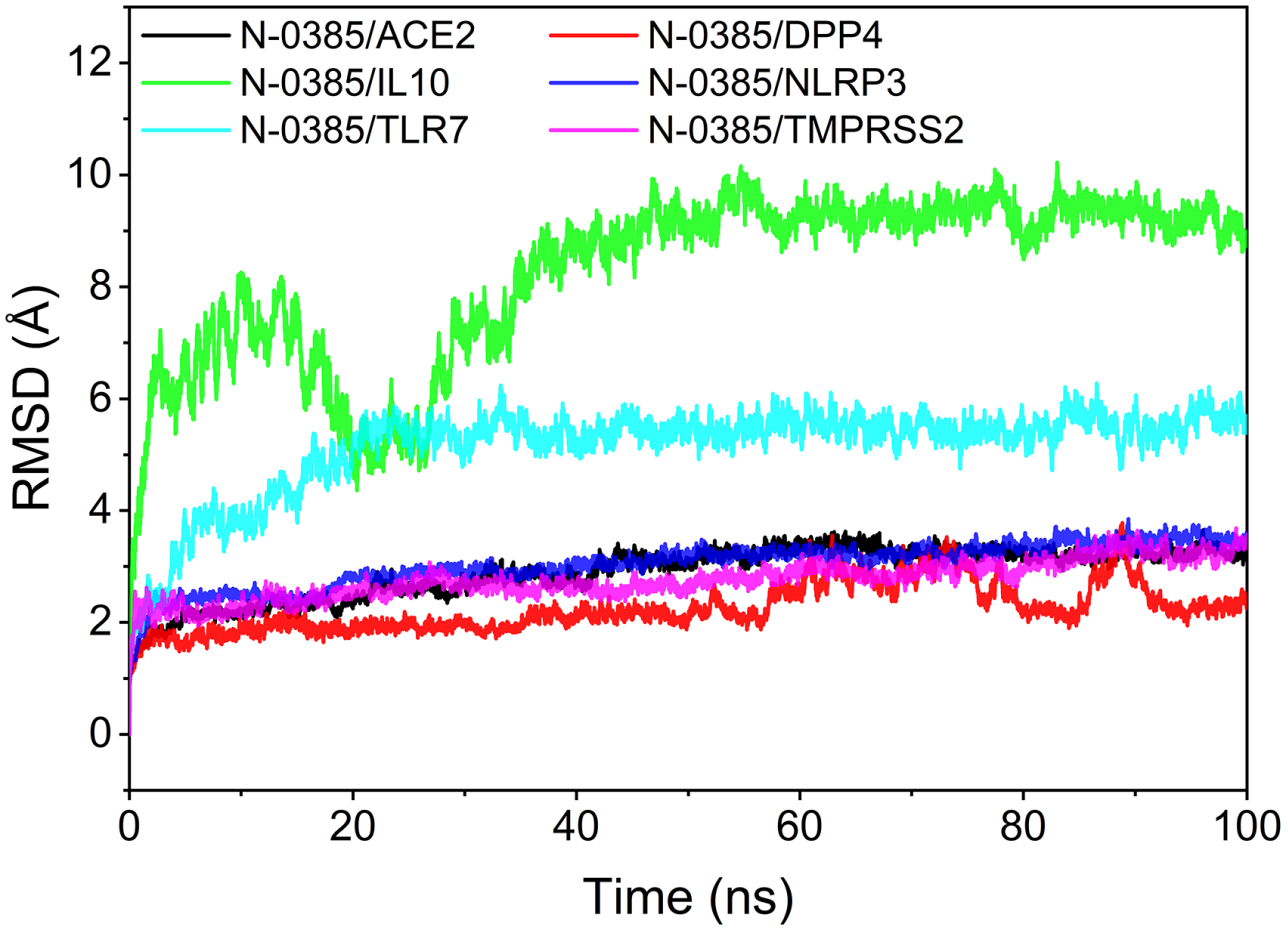
The result of root mean square deviation (RMSD).

The root mean square deviation (RMSD) of molecular dynamics simulations can reflect the motion of the complexes. N-0385/DPP4, N-0385/TMPRSS2, N-0385/ACE2 and N-0385/NLRP3 reached convergence at the beginning of the simulation, and they all preserved stable fluctuations in the subsequent simulations. Although N-0385/TLR7 and N-0385/IL-10 fluctuated sharply at the beginning of the simulation, they both gradually enter a stable state in the later part of the simulation. The overall stability was ranked from high to low as N-0385/DPP4, N-0385/TMPRSS2, N-0385/NLRP3, N-0385/ACE2, N-0385/TLR7 and N-0385/IL-10. The results are shown in Figure 5.

### Results of combining free energy

We calculated the binding energy based on the trajectory of molecular dynamics simulations by the MM-GBSA method, and the binding energy can more accurately reflect the binding mode of small molecules and target proteins. The results of combining free energy of N-0385/ACE2, N-0385/DPP4, N-0385/IL-10, N-0385/NLRP3, N-0385/TLR7 and N-0385/TMPRSS2 were − 12.37 ± 1.68 kcal/mol, −25.83 ± 2.35 kcal/mol, −27.75 ± 2.35 kcal/mol, −18.46 ± 1.84 kcal/mol, −42.73 ± 3.21 kcal/mol and − 30.99 ± 2.37 kcal/mol. The results showed that the small molecule N-0385 and the corresponding proteins possessed some strong binding affinity. The binding free energies of N-0385/TLR7 and N-0385/TMPRSS2 were high, and their binding free energy values were less than −30.0 kcal/mol. The binding energies of these complexes were mainly contributed by Van der Waals energy and electrostatic energy. The experimental results are shown in Table 2.

**Table 2:** Binding free energies and energy components predicted by MM/GBSA (kcal/mol).

System name	Δ*E*_VdW_	Δ*E*_elec_	ΔG_GB_	ΔG_SA_	ΔG_bind_
N-0385/ACE2	−44.78 ± 4.28	−442.35 ± 14.52	481.69 ± 16.90	−6.93 ± 0.44	−12.37 ± 1.68
N-0385/DPP4	−42.69 ± 2.84	−382.48 ± 15.82	405.95 ± 14.17	−6.61 ± 0.34	−25.83 ± 2.35
N-0385/IL-10	−44.44 ± 3.10	−60.49 ± 5.82	83.55 ± 4.12	−6.37 ± 0.24	−27.75 ± 2.35
N-0385/NLRP3	−43.90 ± 5.35	−98.43 ± 15.41	131.09 ± 11.66	−7.21 ± 0.45	−18.46 ± 1.84
N-0385/TLR7	−42.49 ± 2.49	−140.09 ± 10.69	146.94 ± 11.98	−7.08 ± 0.64	−42.73 ± 3.21
N-0385/TMPRSS2	−53.79 ± 3.80	−103.12 ± 13.55	133.01 ± 12.84	−7.10 ± 0.25	−30.99 ± 2.37

Δ*E*_VdW_: Van der Waals energy.

Δ*E*_elec_: electrostatic energy.

ΔG_GB_: electrostatic contribution to solvation.

ΔG_SA_: non-polar contribution to solvation.

ΔG_bind_: binding free energy.

### Hydrogen bond analysis

**Figure 6:**
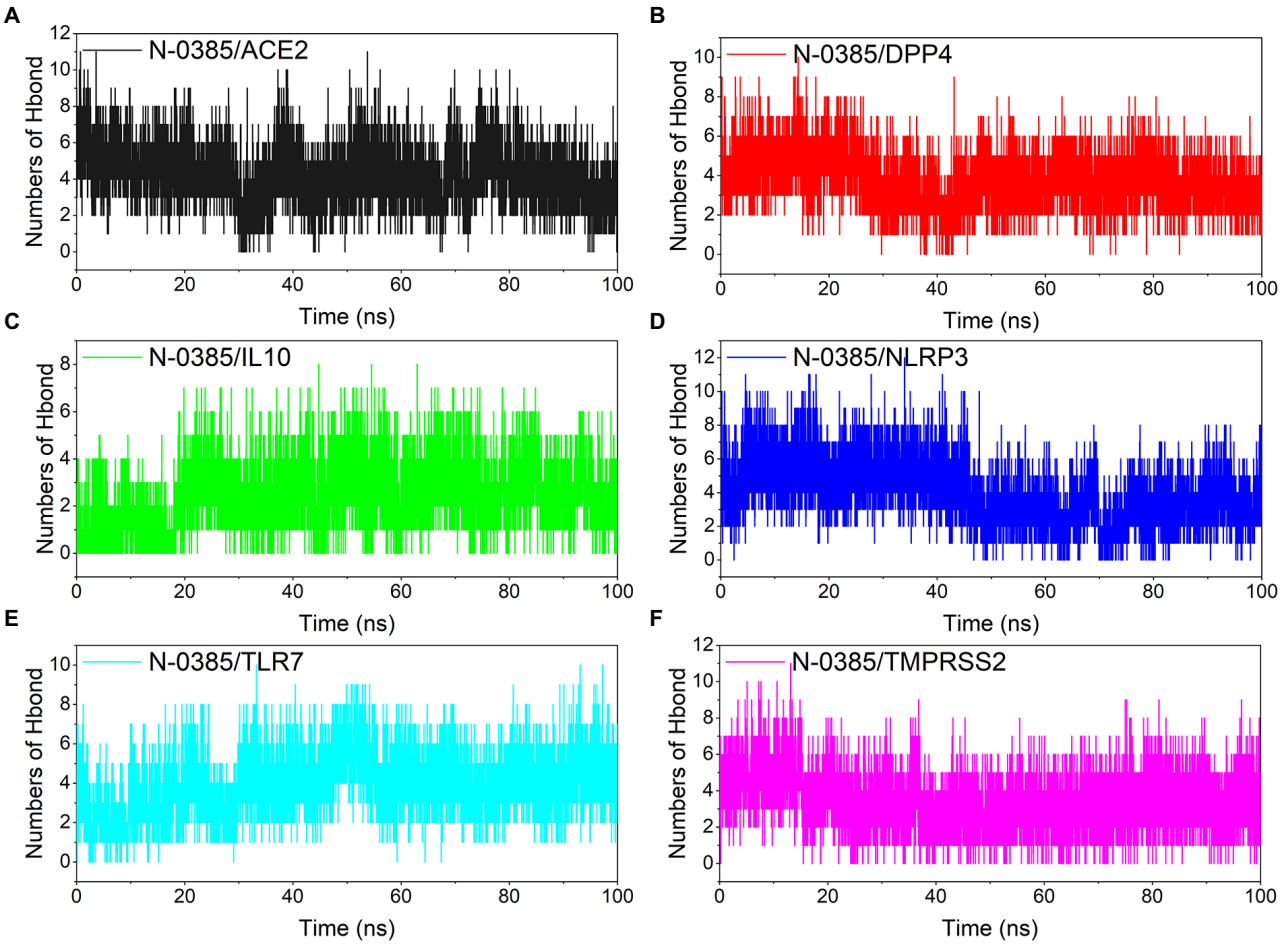
Changes in the number of hydrogen bonds among small molecule ligands and protein receptors in complex system simulations. **(A)** N-0385/ACE2, **(B)** N-0385/DPP4, **(C)** N-0385/IL-10, **(D)** N-0385/NLRP3, **(E)** N-0385/TLR7, **(F)** N-0385/TMPRSS2.

Hydrogen bonding is one of the strongest non-covalent binding interactions, and hydrogen bonding is an important basis for the formation of stable binding small molecule ligands and protein targets. The simulation results showed that all the complexes had a high number of hydrogen bonds during the simulation, and the number of hydrogen bonds was basically maintained above 2. N-0385/ACE2, N-0385/DPP4, N-0385/IL-10 and N-0385/TLR7 even had more than 4 hydrogen bonds in the late stage of simulation, suggesting that hydrogen bonds were very important for the formation of these complexes. The number of hydrogen bonds in N-0385/TMPRSS2 has been maintained at 5–6, and there is an abnormal fluctuation at the beginning of the simulation of N-0385/TMPRSS2. This abnormal fluctuation may be due to a change in the binding state of the receptor protein and the ligand small molecule or the effect of the protein’s own peptide chain. The results are shown in Figure 6.

### Analysis of root mean square fluctuations

**Figure 7:**
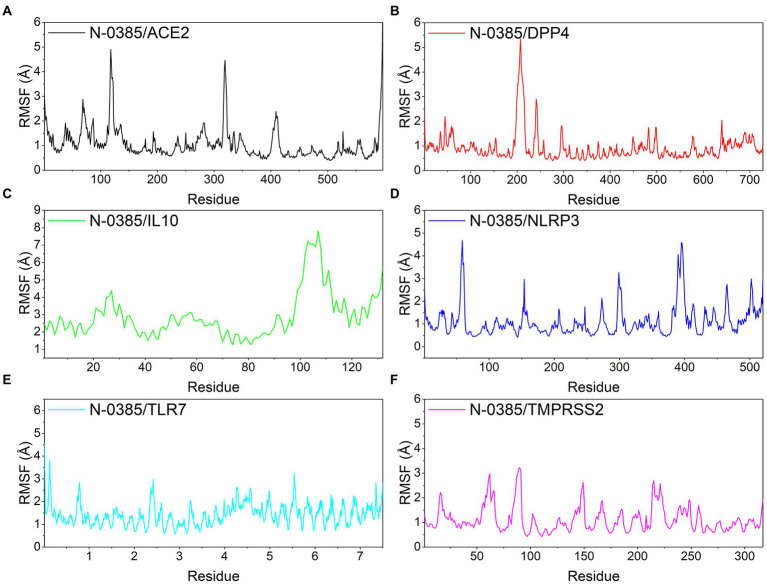
Changes in the stability of protein targets at the residue level. **(A)** N-0385/ACE2, **(B)** N-0385/DPP4, **(C)** N-0385/IL-10, **(D)** N-0385/NLRP3, **(E)** N-0385/TLR7, **(F)** N-0385/TMPRSS2.

Root mean square fluctuations (RMSF) can respond to the flexibility of the protein during molecular dynamics simulation. In general, the flexibility of the protein decreases after drug binding to stabilize the protein. ACE2, DPP4, NLRP3, TLR7 and TMPRSS2 proteins still had low RMSF fluctuations within 3 Å after N-0385 binding, indicating that these proteins have low flexibility and close binding of small molecules. Although the RMSF of the receptor protein IL-10 and the ligand small molecule N-0385 showed large fluctuations after binding, the RMSF was low at the rest of the site except for the two ends of the protein, indicating that the core structure of the protein has good rigidity. The results are shown in Figure 7.

### Analysis of radius of gyration

**Figure 8:**
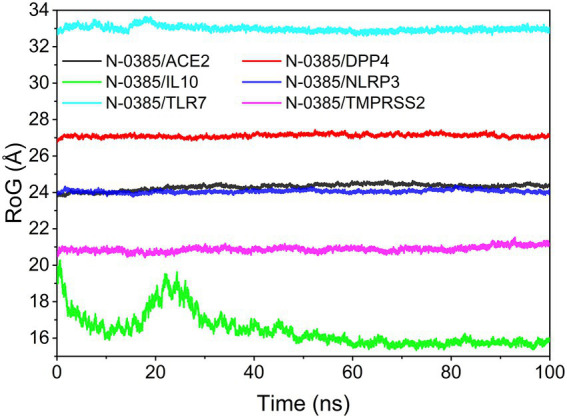
The result of radius of gyration (RoG).

The radius of gyration (RoG) can reflect the degree of compactness of the complex. The RoG simulation results showed that the convergence from the system were N-0385/NLRP3, N-0385/DPP4, N-0385/TMPRSS2, N-0385/ACE2, N-0385/TLR7 and N-0385/IL-10 from the largest to the smallest. The results are shown in Figure 8.

### Analysis of solvent accessible surface area

**Figure 9:**
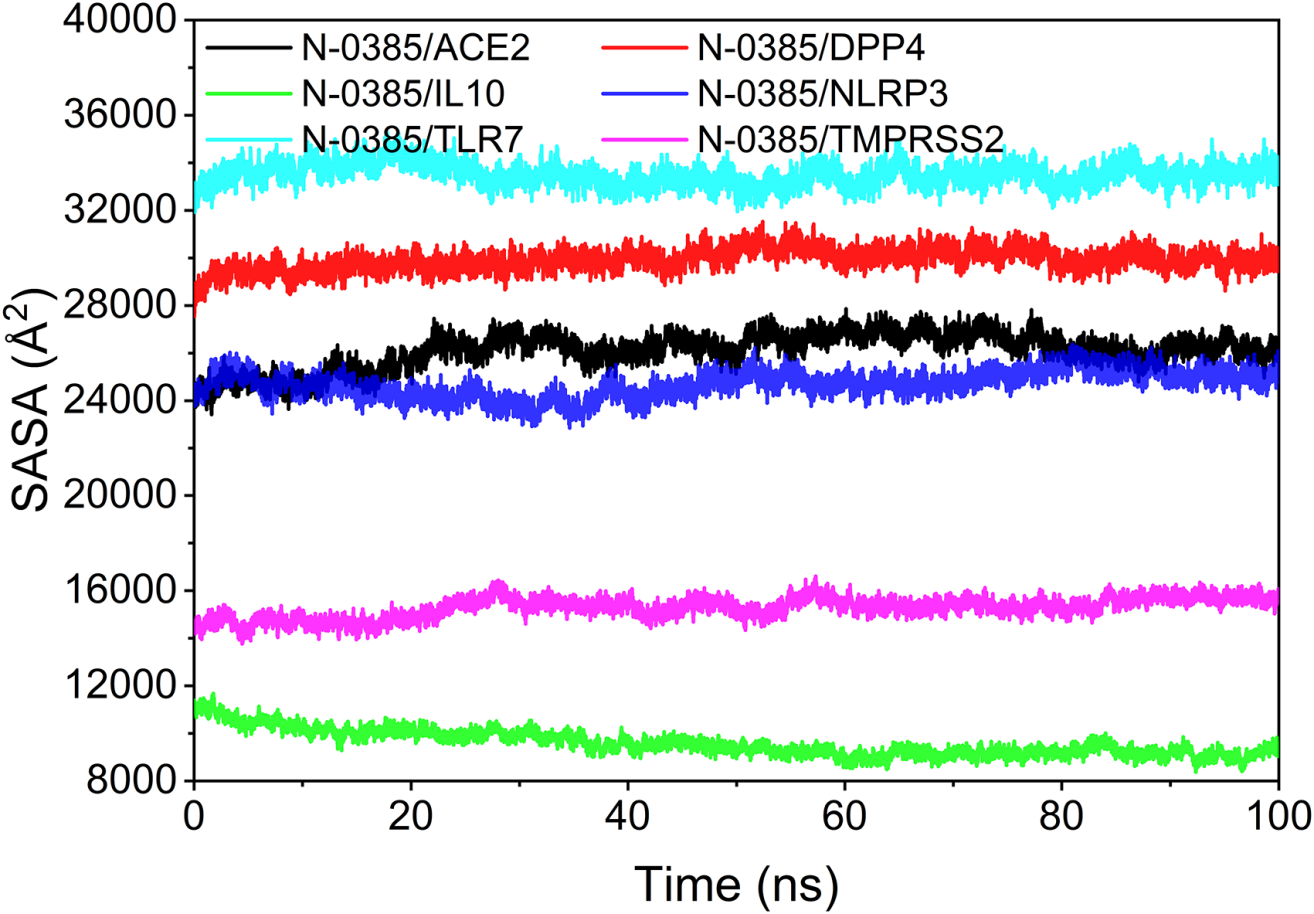
Analysis of solvent accessible surface area (SASA).

The solvent accessible surface area (SASA) indicates the area where the complex can come in contact with the aqueous solution. In addition, the fluctuation of SASA reflects the exposure of the protein surface and the changes that occur in the buried area. Because the systems of the six groups of complexes analyzed in this study are different, the SASA values of different complexes have no comparative value or significance. However, the SASA fluctuations of all complexes were stable indicating that the complexes were able to form tightly bound. The results are shown in Figure 9.

## Discussion

In this study, we investigate the mechanism of N-0385 treatment COVID-19 by molecular docking and molecular dynamics simulation. We speculated that N-0385 may not only inhibit SARS-CoV-2 invasion directly by acting on TMPRSS2, ACE2 and DPP4, but also inhibit the immune recognition process and inflammatory response by regulating TLR7, NLRP3 and IL-10 to prevent SARS-CoV-2 invasion.

Firstly, we conjectured that N-0385 may block hydrolysis and cleavage of SARS-CoV-2 protein by directly acting on TMPRSS2 and ACE2, thereby preventing viral entry into cells. Secondly, N-0385 may reduce viral infection of cells and regulate that *via* metabolism through PDD4, thereby reducing body damage. Finally, N-0385 may reduce the inflammatory response and block the immune recognition process by regulating TLR7, NLRP3 and IL-10, thereby reducing cellular damage and alleviating the disease.

Therefore, these results indicated that N-0385 may act through multiple targets to reduce viral infection and reduce damage consisting of inflammatory responses.

### Analysis of molecular docking and molecular dynamics

Molecular docking can be identified by spatial matching of the drug small molecule N-0385 and the protein macromolecule *in vivo* to each other, and molecular docking can predict their interactions, binding modes and affinities. Molecular dynamics is a powerful tool to analyze structural and dynamic information of biological macromolecular systems. By simulating the microstructural mechanisms and dynamic trajectory behavior of molecules, physicochemical data of molecular systems can be obtained. Molecular dynamics can be used to reveal the microstructural changes of drug small molecule-protein macromolecule interaction systems. Therefore, the results of molecular docking and molecular dynamics can be used to explore the mechanism of N-0385 as it might be used in the treatment of COVID-19.

In this study, the MMGBSA method was used to calculate the binding free energy that is able to reflect the binding stability of ligand small molecules to receptor proteins. The binding free energy results showed that N-0385/TMPRSS2 was −30.99 ± 2.37 kcal/mol, and that N-0385 binds to TMPRSS2 mainly through hydrogen bonding. N-0385/TMPRSS2 converged at the beginning of the RMSD simulation and remained stable in the subsequent simulations. The binding free energy results showed that N-0385/ACE2 and N-0385/DPP4 were − 12.37 ± 1.68 kcal/mol and − 25.83 ± 2.35 kcal/mol. For N-0385/ACE2, the energy analysis showed that hydrophobic interactions were the main contributing energy. And N-0385 was mainly bound to DPP4 through hydrogen bonding. ACE2 and DPP4 still had low RMSF fluctuations within 3 Å after N-0385 binding, indicating that these proteins were low in flexibility and tightly bound to small molecules. The binding free energy results showed −42.73 ± 3.21 kcal/mol, −18.46 ± 1.84 kcal/mol and − 27.75 ± 2.35 kcal/mol for N-0385/TLR7, N-0385/NLRP3 and N-0385/IL-10. The binding energies of these complexes were mainly contributed by Van der Waals and electrostatic energies. N-0385/IL-10 and N-0385/TLR7 formed more than four hydrogen bonds at the late stage of the simulation, indicating that hydrogen bonds were crucial for the formation of these complexes.

### Analysis of the potential mechanism of N-0385 to block SARS-CoV-2 infection of cells

N-0385 may inhibit the invasion of SARS-CoV-2 directly by acting on TMPRSS2, ACE2 and DPP4. N-0385 may block the hydrolysis and cleavage of SARS-CoV-2 protein by acting directly on TMPRSS2 and ACE2, thus preventing the virus from entering the cells, and N-0385 may reduce SARS-CoV-2 infection of cells through PDD4 and regulate organism metabolism, thus reducing organism damage.

Bioinformatics analysis showed that transmembrane serine protease 2 (TMPRSS2) facilitated virus entry into host cells through proteolytic cleavage and activation of viral envelope glycoproteins. KEGG signaling pathway analysis indicated that TMPRSS2 was involved in the mitochondrial immune response to SARS-CoV-2. GO analysis showed that TMPRSS2 was associated with serine-type endopeptidase activity and scavenger receptor activity. Angiotensin-converting enzyme 2 (ACE2) is a functional receptor for the spike glycoprotein of SARS-CoV-2, and plays an important role in the regulation of cardiovascular and renal function and fertility. KEGG signaling pathway analysis included peptide hormone metabolism. GO analysis included metallopeptidase activity and peptide binding. Dipeptidyl peptidase 4 (DPP4) is involved in insulin metabolism and immune regulation. And DPP4 has been shown to be a functional receptor for Middle East respiratory syndrome coronavirus (MERS-CoV). KEGG signaling pathway analysis showed that DPP4 was involved in the metabolism of peptide hormones and the regulation of intestinal insulin. GO analysis suggested that DPP4 was associated with protein homodimerization activity and signaling receptor binding. Protein interaction network analysis showed that TMPRSS2, ACE2 and DPP4 were closely associated with viral invasion targets.

The SARS-CoV-2 spike protein, transmembrane protease serine 2 (TMPRSS2) and human receptor angiotensin-converting enzyme 2 (ACE2) are the major host-pathogen determinants affecting infection (Zhou P. et al., 2020; Senapati et al., 2021). The spike protein is located on the viral outer membrane and the protein has two major functional subunits (a long N-terminal S1 subunit and a relatively short C-terminal S2 subunit). Receptor-Binding Domain (RBD) is located in the S1 subunit of the spiked protein (Li et al., 2005), and TMPRSS2 is located on the surface of type II alveolar cells (Shulla et al., 2011). ACE2 is located on the surface of type II alveolar cells, and it is involved in the regulation of signaling pathways of the renin-angiotensin system and integrin signaling (Shulla et al., 2011; Beacon et al., 2021). In addition, ACE2 can act as a carboxypeptidase, removing a single amino acid from the C-terminus of the substrate (Turner and Hooper, 2002). Study was shown that amino acid-born mutations in the binding sites of spike protein, TMPRSS2 and ACE2 alter the affinity of the proteins, which may affect the structural stability of the complexes (Hussain et al., 2020; MacGowan and Barton, 2020; Yan et al., 2020; Beacon et al., 2021).

The spike protein of the SARS-CoV-2 enters human cells by binding to TMPRSS2 and ACE2. Two distinct modes of cell entry exist for SARS-CoV-2, and the two entry modes differ in the second cleavage. The entry of SARS-CoV-2 into cells occurs by two spike protein cleavages (Senapati et al., 2021). The first occurrence of cleavage takes place when the spike protein and ACE2 are bounden together, there are 20 ACE2 residues interacting with 17 residues from the spike protein RBD, triggering a partial conformational rearrangement in the spike protein and increased sensitivity to hydrolytic digestion of the protein at the junction of the S1 and S2 subunits of the spike protein. This is followed by exposure of the S2’ cleavage site in the S2 subunit (Simmons et al., 2005; Li et al., 2006; Jackson et al., 2022). The second cleavage occurs at the exposed S2’ cleavage site, where multiple arginine-rich sites (Arg667 and Arg797) are recognized and cleaved by two different protein hydrolases, releasing the S2’ subunit. If the target cell TMPRSS2 is not adequately expressed, or if the viral-ACE2 complex does not encounter TMPRSS2, the viral-ACE2 complex is internalized into the endolysosome by histone proteases *via* reticulin-mediated endocytosis to perform hydrolytic cleavage. This cleavage has to occur at the cell surface under acidic conditions and the presence of TMPRSS2 (Jackson et al., 2022). TMPRSS2 can enzymatically cleave a string of hydrophobic amino acids exposed at a site on the S2 subunit of the spiked protein, thereby allowing the virus to rapidly embed into the host cell membrane. Therefore, viruses mediated by TMPRSS2-mediated endocytosis can enter the cell more rapidly (Hoffmann et al., 2020). After cleavage, the fusion peptide is released by intracellular histone protease L15, 16, initiating fusion pore formation (Matsuyama et al., 2010; Glowacka et al., 2011). Subsequently, the unfolded spike protein folds up to fuse the viral outer membrane with the cell membrane thus ensuring that the viral gene can access the cytoplasm.

DPP4 is a serine ectopeptidase, also known as CD26. DPP4 can cleave X-proline dipeptides from the N-terminal end of peptides, and it is expressed in a variety of epithelial and endothelial cells of the systemic vasculature, kidney, lung, small intestine and heart (Solerte et al., 2020a,b). DPP4 is involved in glucose and insulin metabolism and immune regulation. Studies have shown that Middle East respiratory syndrome coronavirus uses DPP4 as its functional receptor (Bassendine et al., 2020). Interestingly, it was demonstrated that there might be a tight interaction between the S1 domain loop of the COVID-19 spike glycoprotein and the CD26 surface (Vankadari and Wilce, 2020). Therefore, like ACE2, DPP4 may act as a SARS-CoV-2 co-receptor into cells (Solerte et al., 2020a,b). Recently, Nádasdi et al. (2022) found that reduced circulating DPP4 activity is associated with severe COVID-19 disease and is a strong prognostic biomarker of COVID-19 mortality. DPP4 inhibitors are commonly used to treat type 2 diabetes. Interestingly, many studies have shown that some DPP4 inhibitors (such as: selegiline and emetine) may have a therapeutic effect on neocoronary, but clinical trials are still needed to confirm their effects (Mikhael et al., 2022; Zhang et al., 2022). In addition, many data show that diabetes is also a factor in causing severe symptoms of COVID-19 (Zhou F. et al., 2020; Godeau et al., 2021). Thus DPP4 may be a potential target for the treatment or prevention of COVID-19 patients with concomitant type 2 diabetes (Iacobellis, 2020).

### N-0385 hinders SARS-CoV-2 invasion by inhibiting recognition processes and inflammatory responses

N-0385 may reduce the inflammatory response and block the immune recognition process by regulating TLR7, NLRP3 and IL-10, thereby reducing cellular damage and alleviating disease.

Bioinformatics analysis suggested that toll like receptor 7 (TLR7) plays an important role in pathogen recognition and innate immune activation, and it recognizes pathogen-associated molecular patterns (PAMPs) expressed on infectious agents and mediates the production of cytokines necessary for effective immunity. KEGG signaling pathway analysis suggested that TLR7 was involved in dendritic cell developmental lineage pathways and in the interaction between immune cells and microRNAs in the tumor microenvironment. GO analysis indicated that NLRP3 was involved in transmembrane signaling receptor activity and double-stranded RNA binding. NLR Family Pyrin Domain Containing 3 (NLRP3) plays a role in the regulation of inflammation, immune response and apoptosis, and NLRP3 can also induce cell death. KEGG signaling pathway analysis included protein metabolism and SARS-CoV-2 activation of the NLRP3 inflammasome. GO analysis included peptidoglycan binding. Interleukin 10 (IL-10) has a potent anti-inflammatory function to which it plays major role in immunomodulatory cytokine, and it can limit excessive tissue destruction caused by inflammation. KEGG signaling pathway analysis suggested that TLR7 was involved in dendritic cell developmental lineage pathways and MIF-mediated glucocorticoid regulation. GO analysis indicated that NLRP3 was involved in cytokine activity and interleukin 10 receptor binding. Protein interaction network analysis showed that TLR7, NLRP3 and IL-10 were closely associated with inflammatory responses and immune regulation.

TLR7 may be involved in SARS-CoV-2 genome recognition through recognition of ssRNA and synthetic oligonucleotides (Khanmohammadi and Rezaei, 2021; Yuan et al., 2021). TLR7 is considered a key cellular sensor of SARS-CoV-2 encoded ssRNA, and it is involved in host resistance and disease pathogenesis of COVID-19 (Salvi et al., 2021). Interestingly, Asano et al. (2021) found that X-linked recessive TLR7 deficiency was a highly exogenous genetic cause of critical COVID-19 pneumonia, and TLR7 and pDC were essential for protective type I IFN immunity against SARS-CoV-2 in the respiratory tract. Some of research have found that the immune response to SARS-CoV-1 *via* TLR7 activation may trigger a cytokine storm (Tang et al., 2016; de Groot and Bontrop, 2020).

IL-10 is a very important class of anti-inflammatory mediators that protects the host from overreacting to pathogens and microbiota, and it can play an irreplaceable role in sterile wound healing, autoimmunity, cancer and homeostasis (Fiorentino et al., 1989; Moore et al., 1990; Saraiva et al., 2020). IL-10 produces a wide range of effects of anti-inflammatory activity by targeting a variety of cells. IL-10 acts mainly through macrophages, and IL-10 triggers a dramatic immunosuppressive response mainly by inhibiting the transcription of cytokines, chemokines, MHCII co-stimulatory and adhesion molecules (Bogdan et al., 1991; Moore et al., 2001; Shouval et al., 2014; Zigmond et al., 2014). Therefore, IL-10 not only regulates the local cytokine microenvironment, but also restricts antigen presentation preventing T cell responses and the spread of inflammation. Studies have found that IL-10 increases ACE2 mRNA expression in lung-derived Calu-3 cells and human umbilical vein endothelial cells (HUVEC), thereby providing cardiopulmonary protection in COVID-19 using a negative feedback mechanism that inhibits inflammation. At the same time, IL-10 promotes viral binding and entry using organismal protective mechanisms to kill pathogens, thereby reducing inflammatory cytokine production and protection from further tissue damage (Albini et al., 2021; Tabares-Guevara et al., 2021).

The NLRP3 inflammasome consists of NLRP3, apoptosis-associated speckle-like protein (ASC) and cysteine aspartase 1 (caspase-1). Activated inflammasomes contribute to the release of mature cytokines, thereby facilitating the development of an innate immune response. It has been shown that the interaction of ACE2 receptors with SARS-CoV-2 spike proteins causes NLRP3 inflammasomes in cells, which may lead to cellular scorching if NLRP3 is over activated (Ratajczak et al., 2020, 2021). NLRP3 inflammasomes trigger inflammatory immune responses *via* intracellular caspase-1, which leads to their ability to release the potent pro-inflammatory cytokines interleukin 1β (IL-1β) and interleukin 18 (IL-18; Franchi et al., 2012; Swanson et al., 2019; Ratajczak et al., 2020; Strollo and Pozzilli, 2020).

## Conclusion

In this study, we investigate the mechanism of therapeutical effect of N-0385 in COVID-19 by molecular docking and molecular dynamics. We speculated that N-0385 may not only directly inhibit SARS-CoV-2 invasion by acting on TMPRSS2, ACE2 and DPP4, but also inhibit the immune recognition process and inflammatory response by regulating TLR7, NLRP3 and IL-10, thus preventing SARS-CoV-2 invasion. N-0385 may have a promising usage in the treatment of COVID-19, for which it blocks SARS-CoV-2 infection *via* multiple targets within inflammatory response. This study renders theoretical basis and new research ideas for N-0385 in the treatment of COVID-19.

## Data availability statement

The datasets presented in this study can be found in online repositories. The names of the repository/repositories and accession number(s) can be found in the article/Supplementary material.

## Author contributions

J-FC, XYY, LX, MW, YZ, and HF contributed to the conception of the study. J-FC, XZ, LX, SC, LZ, CX, PH, YQ, XY, and DL contributed significantly to analysis and manuscript preparation. J-FC, XYY, MW, and SC performed the data analyses and wrote the manuscript. XZ, J-FC, and XH helped to perform the analysis with constructive discussions. All authors contributed to the article and approved the submitted version.

## Funding

This study was supported by “Project of Tibetan Medicine Regional Cooperative Innovation Center (no. 2019xtcx006).”

## Conflict of interest

The authors declare that the research was conducted in the absence of any commercial or financial relationships that could be construed as a potential conflict of interest.

## Publisher’s note

All claims expressed in this article are solely those of the authors and do not necessarily represent those of their affiliated organizations, or those of the publisher, the editors and the reviewers. Any product that may be evaluated in this article, or claim that may be made by its manufacturer, is not guaranteed or endorsed by the publisher.

## Abbreviations

ACE: Angiotensin-converting enzyme
ADFR: AutoDockFR
AMBER: Aamber biomolecular simulation programs
ARDS: acute respiratory distress syndrome
BP: biological process
caspase-1: cysteine aspartase 1
CC: cell component
CCR9: C–C trendy factor receptor 9
COVID-19: 2019 coronavirus disease
DAVID: database for annotation, visualization and integrated discovery
DPP4: dipeptidyl peptidase-4
EGFR: epidermal growth factor receptor
GO: gene ontology
HUVEC: human umbilical vein endothelial cells
IC50: inhibitory concentration
IFN: interferon
IL-10: interleukin 10
IL-18: interleukin 18
IL-1β: interleukin 1β
INS: insulin
KEGG: Kyoto Encyclopedia of Genes and Genomes
MD: molecular dynamics
MERS-CoV: middle east respiratory syndrome coronavirus
MF: molecular function.
MMGBSA: molecular mechanics-generalized born surface area
NLRP3: nucleotide-binding oligomerization domain
PAMPs: pathogen-associated molecular patterns
PME: particle-Mesh-Ewald
PMS2: misty fixing protein 2
PPI: protein–protein Interactions
RBD: receptor-binding domain
RMSD: root mean square deviation
RMSF: root mean square fluctuation
Rog: radius of gyration
S: spike-in
SA: surface area
SASA: solvent accessible surface area
ssRNA: single strand RNA
TLR7: toll-like receptor 7
TMPRSS2: transmembrane serine protease 2
VdW: Van der Waals force
